# Somatic mutations predict prognosis in myelodysplastic syndrome patients with normal karyotypes

**DOI:** 10.1038/s41392-021-00606-3

**Published:** 2021-07-26

**Authors:** Xiangzong Zeng, Yu Zhang, Ke Zhao, Lingling Zhou, Ya Zhou, Li Xuan, Rui Cao, Jun Xu, Min Dai, Qifa Liu

**Affiliations:** Department of Hematology, Nanfang Hospital, Southern Medical University, Guangzhou, China

**Keywords:** Cancer genetics, Prognostic markers

**Dear Editor**,

Myelodysplastic syndromes (MDSs) are a group of clonal myeloid stem cell disorders characterized by varying degrees of cytopenias, cytogenetic and molecular genetic abnormalities, and a predisposition to acute myeloid leukemia (AML). The treatments for MDS mainly consist of cytoreductive treatment, such as traditional AML-like chemotherapy, hypomethylating agents (HMAs), allogeneic hematopoietic stem cell transplantation (allo-HSCT) and immunoregulation according to risk stratification. Identification of individualized prognostic risk factors is important for guiding optimal treatment. The International Prognostic Scoring System (IPSS) and its revised version (IPSS-R), have been developed to stratify MDS, which mainly depend on peripheral blood parameters, cytomorphology, and conventional cytogenetics. However, more than half of patients have normal karyotypes, and a few patients do not acquire cytogenetic information in clinical practice. Increasing evidence indicates that somatic mutations are also important prognostic risk factors for MDS.^1^ Here, we investigated the impact of somatic mutations on prognosis in MDS patients with normal karyotypes.

A total of 366 consecutive patients with newly diagnosed MDS between October 2012 to June 2019 were identified, and 304 were enrolled in this study, with the exception of 62 patients who were absent in genetic information. There were 124 females and 180 males, with a median age of 51 (range: 15–87) years. They were divided into two groups according to cytogenetics: normal and aberrant karyotype groups. The patients’ demographics and baseline characteristics at diagnosis are detailed in Supplementary Table S1.

One hundred and forty (46.1%) patients had aberrant karyotypes (Supplementary Fig. S1). The most frequent abnormalities were complex karyotypes (Supplementary Table S2.). Somatic mutations were identified in 113 of the 127 selected genes, and the next-generation sequencing panel is shown in Supplementary Table S3. Two hundred and eighty-one patients (92.4%) harbored at least one mutation, along with 148 (90.2%) cases in the normal group and 133 (95.0%) cases in the aberrant group (*P* = 0.118). The number of mutations in the normal was greater than that in aberrant groups, with means of 3.87 ± 2.87 and 3.12 ± 2.14 (*P* = 0.011), respectively. The genomic architecture of the whole cohort (mutation frequency ≥2%) is detailed in Supplementary Fig. S1. The distribution of mutations in different groups of IPSS-R is shown in Supplementary Table S4. The frequencies of mutations detected in >5% of the whole cohort were compared, and the results showed that ASXL1, CD101, KDM6A, SH2B3, and IL-3RA mutations were more common in the normal group, while TET2 and TP53 were more common in the aberrant group (all *P* < 0.05) (Supplementary Fig.S2). Correlations were analyzed between genetic abnormalities, and positive correlation was found between complex karyotypes and TP53 mutations. SH2B3 mutations showed positive correlations with FAT1 and CD101 mutations (Supplementary Fig. S3a, b).

Ninety-five patients received cytoreductive treatment without allo-HSCT, including 63 HMAs, 30 chemotherapy combined with HMAs and 2 traditional AML-like chemotherapy, 126 allo-HSCT, including 45 without cytoreduction and 81 with cytoreduction pre-transplantation, 14 immunoregulatory, 66 supportive care, and 3 patients abandoned treatment. There were more patients who received supportive care treatment in the normal than aberrant groups (*P* = 0.001) since there were more low-risk patients in the normal group (*P* < 0.0001) (Supplementary Table S1). The other treatments were not significantly different between the two groups (all *P* > 0.05) (Supplementary Table S1). Of the 95 patients underwent cytoreduction, there were 51 patients in the normal and 44 patients in aberrant groups, with median cycles of 4 (range: 1–10) and 5 (range: 1–12), respectively (*P* = 0.240). The response of cytoreduction is shown in Supplementary Table S5a. Compared to wild types, the overall response rate (complete response (including marrow CR) + partial response+hematological improvement) was lower in patients with TP53 mutations, while it was higher with EP300 mutations (Supplementary Table S5b).

With a median follow-up of 24 (1–86) months post-diagnosis, 62 patients developed leukemia, including 35 cases in the normal group and 27 cases in the aberrant group (*P* = 0.657), with 12 (range: 2–26) months and 9 (range: 1–20) months at median transformation time, respectively (*P* = 0.111). Cox regression analysis showed that IPSS-R (HR 1.53; *P* = 0.021) and DDX18 (HR 2.67; *P* = 0.043) mutations were risk factors for leukemia transformation in the whole cohort, while TET2 (HR 5.55; *P* = 0.034) mutations in the normal karyotype group and RUNX1 (HR 5.43; *P* = 0.012), U2AF1 (HR 17.53; *P* = 0.006) and DDX18 mutations (HR 9.03; *P* = 0.016) in the aberrant karyotype group were risk factors (Supplementary Table S6). Patients in the high/very high groups had a higher risk of leukemia transformation than those in the very low/low/intermediate groups (Supplementary Table S7).

During follow-up, 175 patients survived, and 129 died. The causes of death are shown in Supplementary Table S8. The 2-year overall survival (OS) were 64.8% and 38.3% in the normal and aberrant groups (*p* < 0.0001), and they were 65.0% and 47.1% in patients with allo-HSCT and patients without allo-HSCT (*p* = 0.005), respectively (Supplementary Fig. S4). Multivariable analysis showed that age (HR 1.02; *P* = 0.027), IPSS-R (HR 1.80; *P* < 0.0001), TP53 (HR 2.36; *P* < 0.0001), and DNMT3A (HR 1.83, *P* = 0.044) were risk factors, while allo-HSCT (HR 0.50; *P* = 0.001) was a protective factor for OS in the whole cohort. For subgroup analysis, age (HR 1.04; *P* = 0.012; HR 1.02; *P* = 0.036, respectively), IPSS-R (HR 1.54; *P* = 0.005; HR 1.80; *P* < 0.0001, respectively), TP53 mutation (HR 2.49; *P* = 0.030; HR 2.13; *P* = 0.005, respectively), and allo-HSCT (HR 0.52; *P* = 0.040; HR 0.37; *P* < 0.0001, respectively) retained prognostic significance in the normal and aberrant groups, while DNMT3A, FAT1, and IL-7R mutations were unfavorable factors for OS only in the normal group (HR 3.32; *P* = 0.006) (HR 2.32; *P* = 0.019) (HR 4.35; *P* = 0.002), respectively (Supplementary Table S6). Types of mutations that included in the multivariable COX analysis were detailed in Supplementary Table S9.

In the normal group, the 2-year OS were 43.8%, 48.3% and 41.7% and 68.1%, 69.3% and 67.7% in the patients with or without DNMT3A, FAT1 and IL-7R mutations, respectively (*P* < 0.0001, *P* = 0.026, *P* = 0.001, respectively), while in the aberrant group, they were 42.9%, 25% and 40% and 37.9%, 40.6% and 37.6%, respectively (*P* = 0.932, *P* = 0.583, *P* = 0.441, respectively) (Fig. 1a–f). The patients with TP53 mutations had a worse prognosis in both the normal and aberrant groups (*P* = 0.026, *P* < 0.0001, respectively), and allo-HSCT might improve the OS of these patients (*P* = 0.034, *P* = 0.012, respectively) (Fig. 1g–j). However, the OS of patients with DNMT3A, FAT1, or IL-7R mutations might not be improved by allo-HSCT in the normal group (*P* = 0.954, *P* = 0.600, *P* = 0.923, respectively) (Supplementary Fig. S5).

**Fig. 1:**
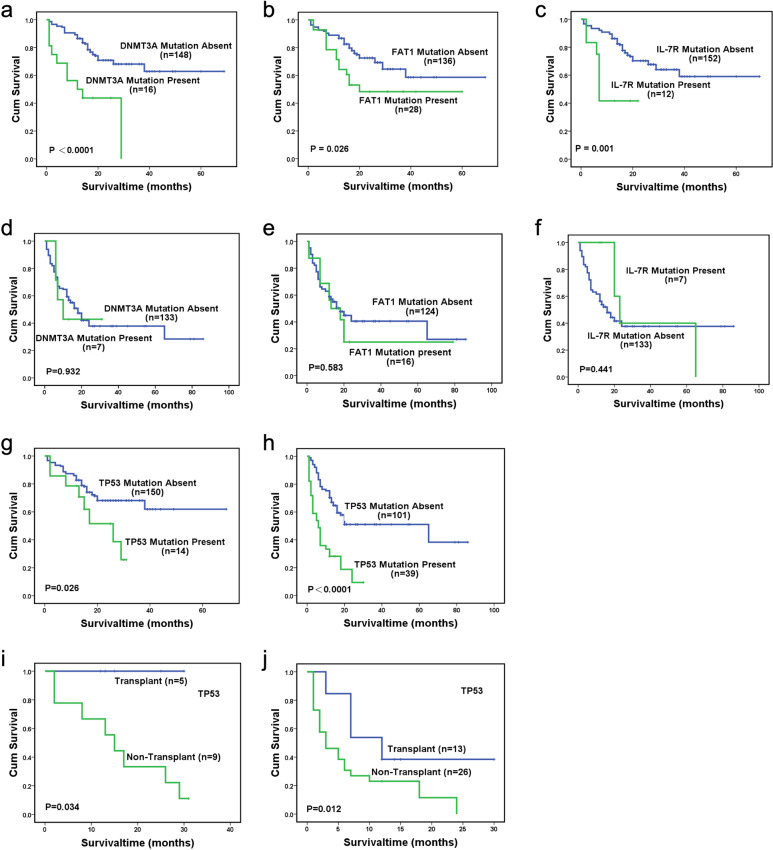
Overall survival (OS) of MDS patients. **a**, **b**, **c**, **g** Classified by DNMT3A, FAT1, IL-7R and TP53 mutations status in the normal karyotype group; **d**, **e**, **f**, **h** Classified by DNMT3A, FAT1, IL-7R, and TP53 mutations status in the aberrant karyotype group; **i** OS of patients with TP53, classified by treatment (transplant or non-transplant) in the normal karyotype group; **j** OS of patients with TP53, classified by treatment (transplant or non-transplant) in the aberrant karyotype group

In summary, we identified four mutated genes—TP53, DNMT3A, FAT1, and IL-7R mutations—that were associated with shorter survival in MDS patients with normal karyotypes, while only TP53 mutation was found to be adverse in aberrant group. TP53 mutation has been known as an unfavorable factor for OS. Our results showed that allo-HSCT might improve the OS of patients with TP53, which was consistent with a previous report.^2^ Some studies had suggested that DNMT3A was an adverse factor, but whether allo-HSCT can overcome it remains unclear in MDS.^1,3^ Our results indicated that OS of patients with DNMT3A was not improved by allo-HSCT. FAT1 mutation was related to poor prognosis in solid tumors,^4^ and IL-7R mutation had a lower survival in acute lymphoblastic leukemia.^5^ The prognostic significance of FAT1 and IL-7R, and whether allo-HSCT can improve the outcomes in MDS with these mutations have not been reported. The current study is the first to report that FAT1 and IL-7R mutations are associated with poor prognosis in MDS patients with normal karyotypes, and allo-HSCT might not overcome their adverse effects, but these need to be validated with a large sample size.

The value of the present study is limited by the relatively small number of patients and the heterogeneity of the treatment regimens. However, different treatment options for MDS are inevitable in clinical practice. Furthermore, because patients were enrolled in the present study only depending on sample availability, our findings might have some selection bias.

## Supplementary information

Supplementary materials

## Data Availability

All data generated or analyzed during this study are included in this manuscript or supplementary materials.
