# Supplementary material for: Somatic mutations predict prognosis in myelodysplastic syndrome patients with normal karyotypes
Source: Signal Transduct Target Ther. 2021 Jul 26;6:274. doi: 10.1038/s41392-021-00606-3 (PMC8310889; doi:10.1038/s41392-021-00606-3)
Supplement: Supplementary file 1 — Supplementary materials [file 41392_2021_606_MOESM1_ESM.docx]

Supplementary Materials for

Somatic mutations predict prognosis in myelodysplastic syndrome patients with normal karyotypes

Xiangzong Zeng#, Yu Zhang#, Ke Zhao, Lingling Zhou, Ya Zhou, Li Xuan, Rui Cao, Jun Xu, Min Dai *, Qifa Liu *

#Xiangzong Zeng and Yu Zhang contributed equally to this work.

Correspondence to: Qifa Liu Email: [liuqifa628@163.com](mailto:liuqifa628@163.com) or

Min Dai Email: berrydai2003@aliyun.com

**This PDF file includes:**

Materials and Methods

Figures. S1 to S5

Tables S1 to S9

Materials and Methods

Study design and patients

This retrospective study cohort examined all MDS patients at diagnosis from October 2012 to June 2019 at our institution. Medical records of all patients were reviewed for demographics, cytogenetic and molecular genetic data, IPSS-R, the treatment-related parameters and so on. Patients were enrolled in this study if they were:1) aged ≥16 years; 2) cytogenetics and next-generation sequencing (NGS) data. Details of follow-up data came from medical records and telephone follow-up. All patients were followed up to the date they died or at least one year after diagnosis. Diagnostic criteria and histological subtypes were according to the World Health Organization (WHO), [^1^](#_ENREF_1) while risk stratification was categorized according to the IPSS-R[^2^](#_ENREF_2) and response evaluation was according to International Working Group (IWG).[^3^](#_ENREF_3) Cytogenetic abnormalities were categorized according to the MDS Cytogenetic Scoring System, and complex karyotypes were defined as three or more chromosomal abnormalities.^2^ The study was approved by the Ethics Committee of Nanfang Hospital, Southern Medical University(Ethical approval No. NFEC-2017-195) and conducted in accordance with the Helsinki Declaration of 1975, as revised in 2008. Informed consent was obtained from all patients before the study.

Cytogenetics and mutation assessment

Bone marrow specimens were used for cytogenetic analysis by conventional chromosome banding techniques and/or fluorescence in situ hybridization. Twenty metaphases were analyzed, and the results were reported using the current International System for Human Cytogenetic Nomenclature. A genomic panel of 127 gene targets were detected by [NGS](C:/Users/Administrator/AppData/Local/youdao/dict/Application/7.5.2.0/resultui/dict/javascript:;) using bone marrow specimens at the time of diagnosis. NGS was performed using a specific multiple-gene panel to explore the mutational status of selected regions (amplicons 1125; amplicon range:125-375bp; coverage 99.16%). By the Ion Torrent PGM System, the sequencing was performed with the Ion 318 Chip Kit. The reference sequence is the human genome GRCM7.

Statistical analysis

Comparison of numerical variables between groups was carried out using a Student's t test or nonparametric approach (Mann-Whitney test). Comparison of the categorical variables and correlation analysis in different groups was performed with either Fisher’s exact test (2 × 2 tables) or the X^2^ test (larger tables). Overall survival (OS) was defined as the time between diagnosis and deaths (from any cause) or last follow-up (for censored observations). Curves were constructed for OS using the Kaplan-Meier method and compared using a log-rank test. Univariable and multivariable survival analysis were performed using Cox proportional hazards regression. When *P* values less than 0.1, variables were included in the multivariable analyses. *P* values were two sided and considered signifificant if less than 0.05. Analyses were performed using SPSS 21.0.

References

1 Arber, D. A. *et al.* The 2016 revision to the World Health Organization classification of myeloid neoplasms and acute leukemia. *Blood*. **127**, 2391-2405, (2016).

2 Greenberg, P. L. *et al.* Revised international prognostic scoring system for myelodysplastic syndromes. *Blood*. **120**, 2454-2465, (2012).

3 Cheson, B. D. *et al.* Clinical application and proposal for modification of the International Working Group (IWG) response criteria in myelodysplasia. *Blood*. **108**, 419-425, (2006).

Figure S1.

**a**


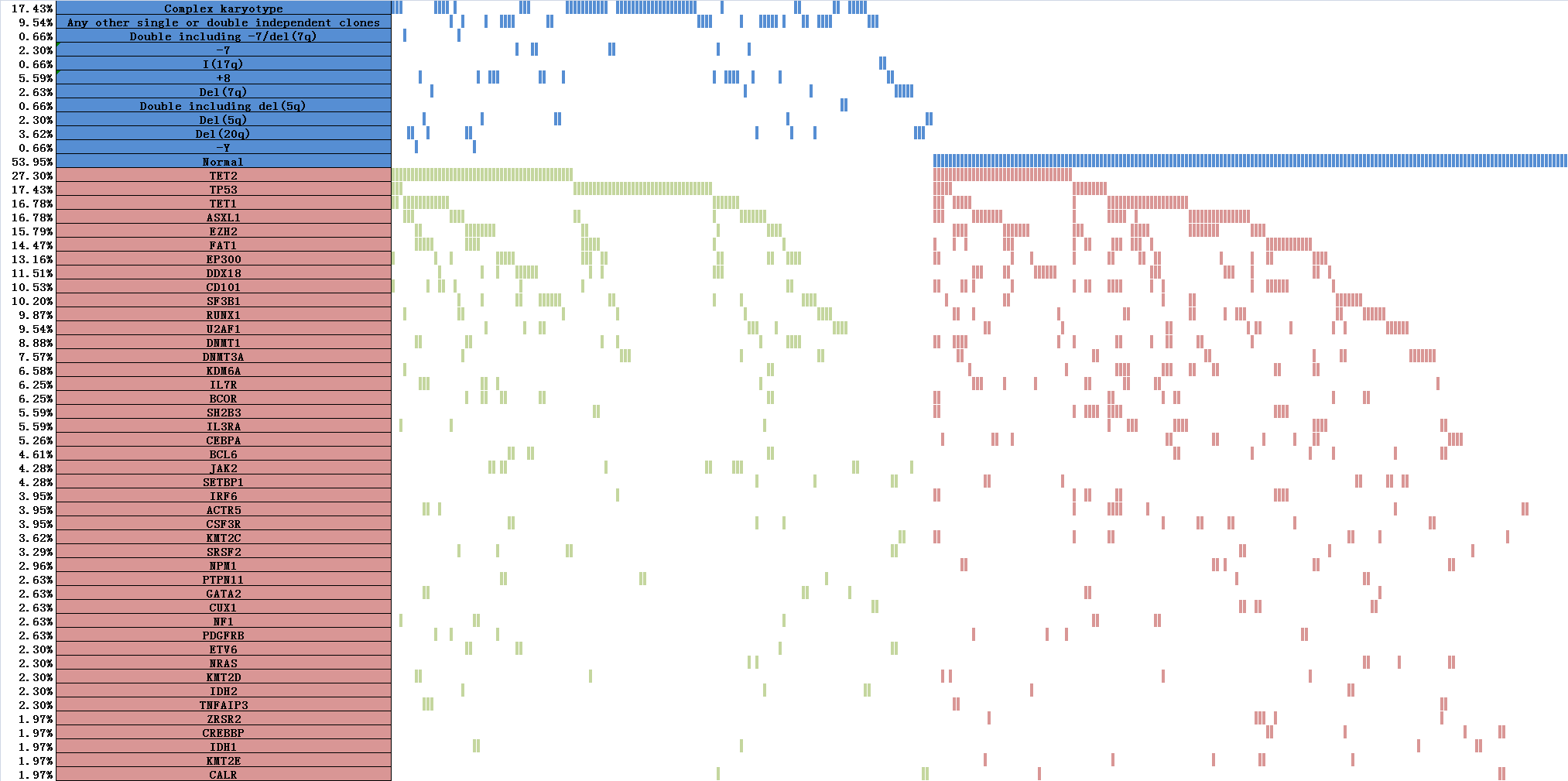


**b**

**Supplementary Figure S1. The genomic architecture of the whole cohort (mutation frequency ≥2%).** (a) Mutation landscape with respects of karyotypes. (b) Frequencies of mutations.

Figure S2.

**Supplementary Figure S2.** The differences of genetic mutation frequency between the normal and aberrant karyotype groups.

Figure S3.

**a**

**
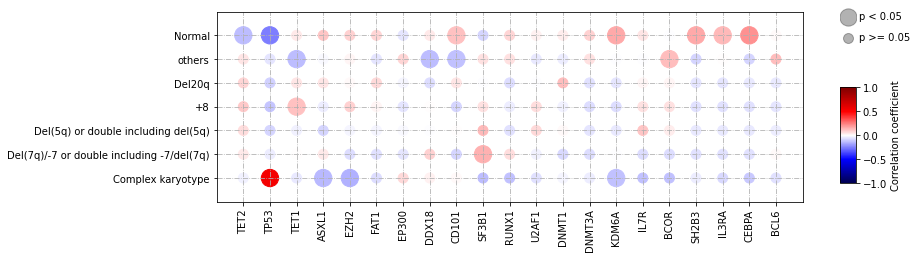
**

**b**


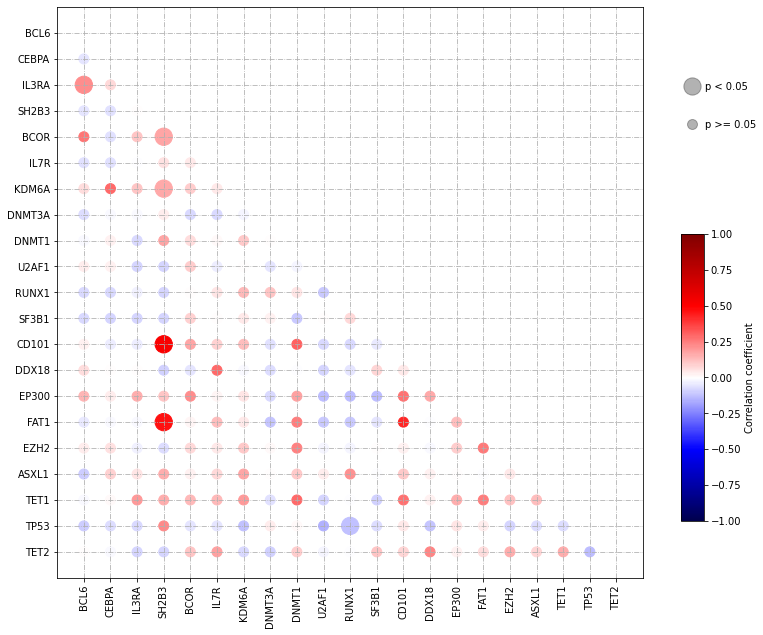


**Supplementary Figure S3. Correlations between genetic lesions.** (a) Comparison of karyotypes and mutation; (b) Comparison of mutation status between major genes (mutation frequency ≥5%).

Figure S4.

| **a** | **b** |
| --- | --- |
| 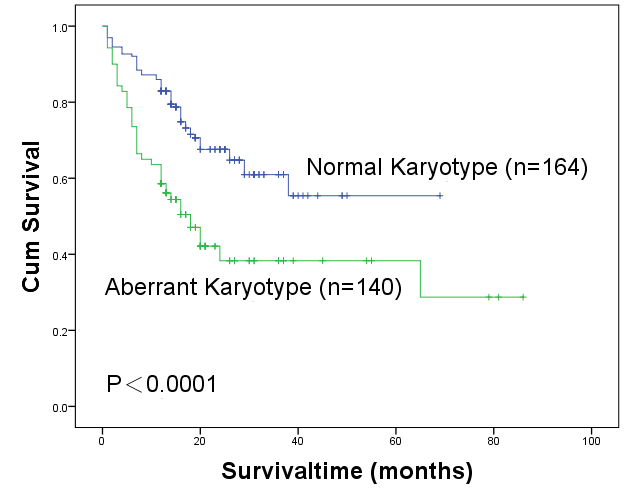 | 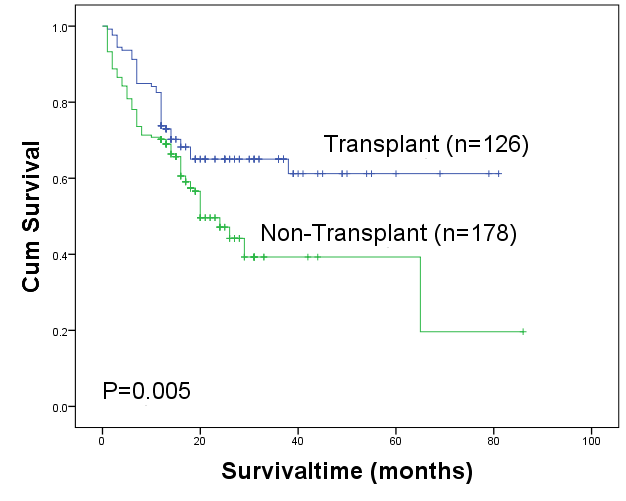 |

**Supplementary Figure S4. Overall survival (OS) of MDS patients** (a) classified by karyotypes (normal or aberrant); (b) classified by treatment (transplant or non-transplant).

Figure S5.

| **a**  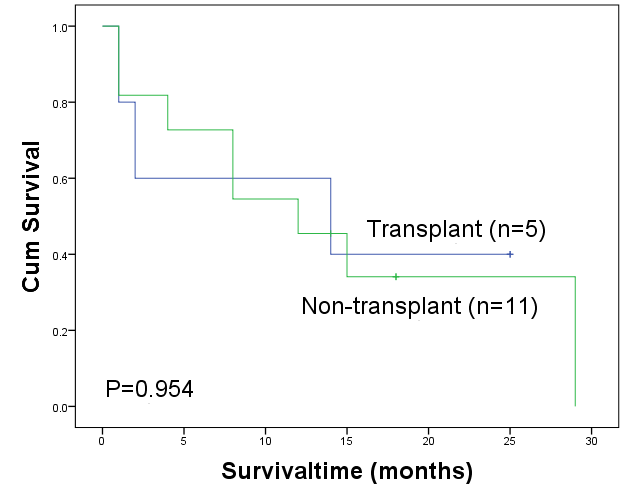  DNMT3A | **b**  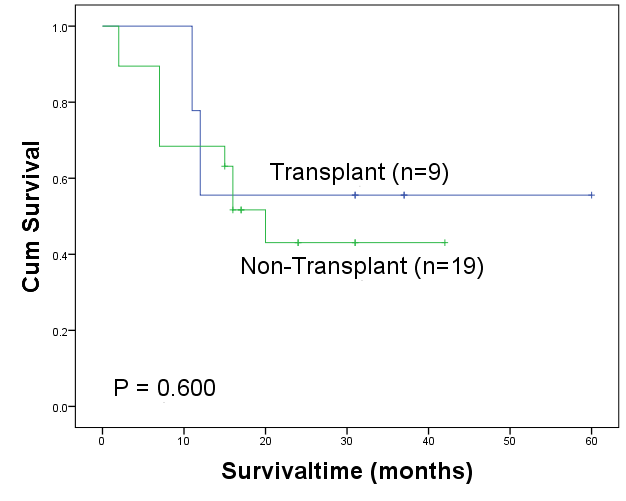  FAT1 | **c**  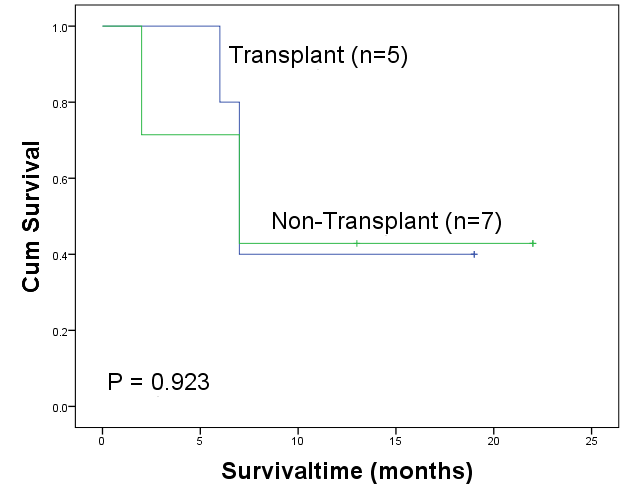  IL-7R |
| --- | --- | --- |
| **d**  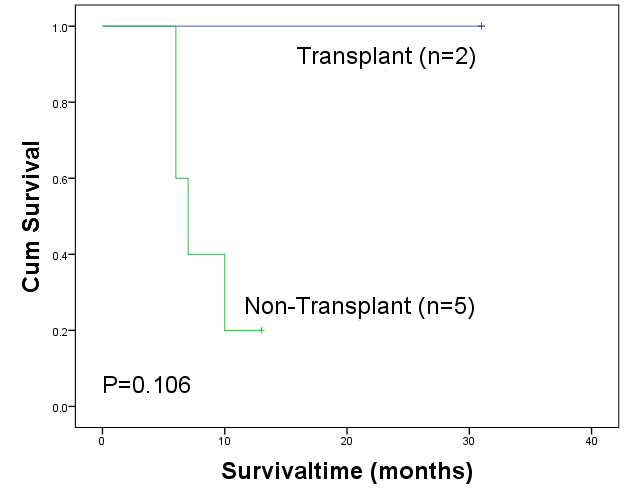  DNMT3A | **e**  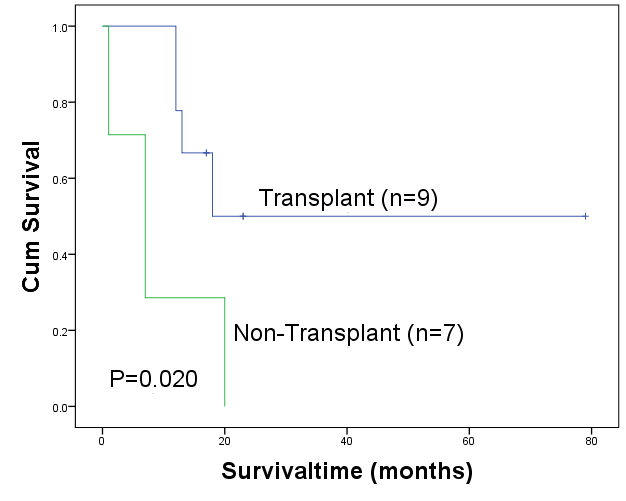  FAT1 | **f**  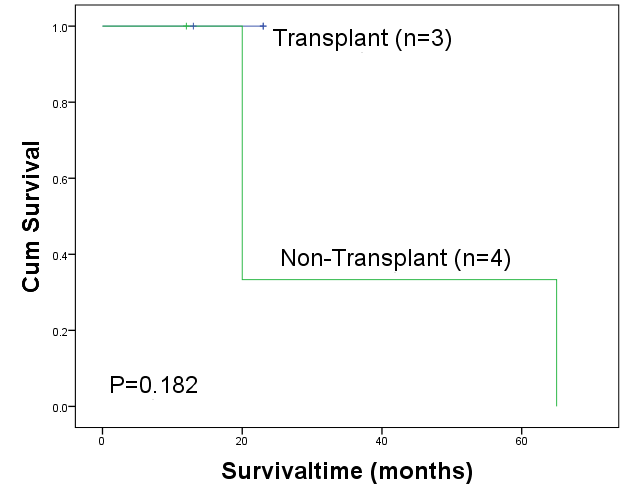  IL-7R |

**Supplementary Figure S5. OS of patients with DNMT3A, FAT1 or IL-7R mutations classified by treatment (transplant or non-transplant).** (a; b; c) in the normal karyotype group;(d; e; f) in the aberrant karyotype group.

Table S1. Patients’ demographic and clinical characteristics (N=304)

| **Parameter** | **Normal Karyotypes (N=164)** | **Aberrant karyotypes**  **(N=140)** | **P** |
| --- | --- | --- | --- |
| Age (years), median, range | 52 (15-87) | 50(18-76) | 0.897 |
| **Gender, no. (%)** |  |  | **<0.0001** |
| Male sex | 78(47.6%) | 102(72.9%) |  |
| Female sex | 86 (52.4%) | 38(27.1%) |  |
| Median WBC (range), ×10^9^/L | 2.66(0.27-156.40) | 2.49(0.42-52.33) | 0.478 |
| Median NE (range), ×10^9^/L | 0.98(0.02-89.40) | 0.78(0.04-7.55) | 0.009 |
| Median HGB (range), g/L | 69 (30-135) | 68(30-210) | 0.888 |
| Median PLT (range), ×10^9^/L | 66(1-519) | 57(2-469) | 0.567 |
| **MDS subtypes (WHO, 2016)** |  |  | **<0.0001** |
| MDS-SLD | 15 (9.1%) | 2(1.4%) | 0.004 |
| MDS-MLD | 37(22.6％) | 22(15.7%) | 0.132 |
| MDS-RS-SLD | 0 (0.0%) | 2(1.4%) | 0.211 |
| MDS-RS-MLD | 10 (6.1%) | 14(10.0%) | 0.209 |
| MDS with isolated 5q-deletion | 0 (0.0%) | 7(5.0%) | 0.004 |
| MDS-EB-1 | 29 (17.7%) | 41(29.3%) | 0.017 |
| MDS-EB-2 | 70 (42.7%) | 46(32.9%) | 0.079 |
| MDS-U | 3 (1.8%) | 6(4.3%) | 0.310 |
| **IPSS-R risk group, no. (%)** |  |  | **<0.0001** |
| Very low | 6 (3.7%) | 1(0.7%) | 0.129 |
| Low | 33(20.1%) | 5(3.6%) | <0.001 |
| Intermediate | 39 (23.8%) | 27(19.3%) | 0.343 |
| High | 65 (39.6%) | 41(29.3%) | 0.059 |
| Very high | 21 (12.8%) | 66(47.1%) | <0.0001 |
| **Treatment, no. (%)** |  |  | **0.002** |
| Allogeneic HSCT | 60(36.6%) | 66(47.1%) | 0.063 |
| Cytoreductive therapy | 51(31.1%) | 44(31.4%) | 0.951 |
| Immunoregulatory | 5(3.0%) | 9(6.4%) | 0.161 |
| Supportive care | 48(29.3%) | 18(12.9%) | 0.001 |
| Abandoned treatment | 0(0.0%) | 3(2.1%) | 0.097 |
| Leukemia transformation, no. (%) | 35 (21.3%) | 27 (19.3%) | 0.657 |
| Transformation time(months), median | 12 (2-26) | 9 (1-20) | 0.111 |

*WBC,* white blood cell count; *NE,* neutrophils; *HGB,* hemoglobin; *PLT,* platelet; *MDS-SLD,* MDS with single-lineage dysplasia; *MDS-MLD,* MDS with ring sideroblasts with multilineage dysplasia; *MDS-RS-SLD,* MDS with ring sideroblasts with single-lineage dysplasia; *MDS-RS-MLD,* MDS with ring sideroblasts with multilineage dysplasia; *MDS-EB*, MDS with excess of blasts; *MDS-U*, MDS unclassifiable; *HSCT*, hematopoietic stem cell transplantation.

Table S2. Chromosomal abnormalities included in the complex karyotypes.

| Numerical abnormality | | -7;-5;-17;-18;-22;-1;-2;-3;-4;-6;-8;-9;-10;-11;-12;-13;-14;-15;-16;-19;-20;-21;-Y. |
| --- | --- | --- |
|  |  | +8;+9;+19;+11;+21;+1;+3;+4;+5;+6;+13;+14;+15;+17;+18;+21;+22;+X;+Y;+mar1;+mar2;+mar3;+mar4. |
| Structural abnormality | Deletion | del(5)(q23q32);del(5)(q31q35);del(5)(q22q31);del(5)(q13q33);del(5)(q12q33);del(5)(q22q35);del(5)(q15q33);del(5)(q15q35);del(5)(q22q35);del(5)(q14);del(7)(q22);del(7)(q22q32);del(7)(p11);del(20)(q11.2q13.3);del(20)(q11q13);del(20)(q11);del(12)(q24);del(12)(p11);del(12)(p12);del(11)(q23);del(1)(p22);del(1)(p13);del(3)(q21);del(3)(p26);del(4)(q27q31);del(4)(q25);del(2)(p21);del(8)(q24);del(9)(q22);del(13)(q22);del(17)(p11). |
|  | Addition | add(3)(q11);add(3)(q29);add(3)(p13);add(3)(p26);add(4)(q32);add(7)(q36);add(9)(q34);add(9)(p13);add(9)(p11);add(10)(p13);add(11)(p15);add(12)(p11);add(14)(p11);add(21)(q22). |
|  | Derivation | der(1)inv(1)(p22p36);der(1)t(1;19)(q23;p13);der(1);der(3);der(3)t(3;19)(p13;p13);der(5)t(3;5)(q11;p15);der(5);der(7)t(5;7)(q11;q11);der(7)t(7;17)(q22;q12);der(11);der(12)t(1;12)(p11.2;q31);der(12)t(12;15)(p11;q11);der(14)t(12;14)(q11;p11.2);der(15)t(1;15)(p11;q11);der(15)t(14;15)(q10;q10);der(15)t(15;17)(p11;q11);der(19)t(19;21)(p11;q11);der(19);der(19)t(5;19)(q23;p13.3);der(21)t(3;21)(p13;p11);der(21)t(21;22)(q10;q10);der(22)t(1;22)(p22;q13). |
|  | Insertion | ins(3;15)(p21;q26q22);ins(1;14)(p22;p11q32);ins(12;16)(q13;p11p13). |
|  | Translocation | t(13;18)(q10;q10);t(9;16)(q12;p11). |
|  | Inversion | inv(3)(p13q21);inv(16)(p13q22). |
|  | Duplication | dup(1)(q21q32). |
|  | Isodicentric | idic(20)(p11). |

Table S3. The NGS panel for targeted sequencing.

| ABCA12 | BLM | CYP3A4 | FGFR3 | JAK3 | MYD88 | SETBP1 | TERT |
| --- | --- | --- | --- | --- | --- | --- | --- |
| ABL1 | BRAF | DDX18 | FLT3 | KDM2B | NF1 | SETD2 | TET1 |
| ABL2 | CALR | DDX41 | GATA1 | KDM5A | NOTCH1 | SF1 | TET2 |
| ACTR5 | CBL | DIS3 | GATA2 | KDM6A | NOTCH2 | SRSF2 | TLX3 |
| ADAMTS13 | CCND3 | DNM2 | GATA3 | KMT2A | NPM1 | STAG1 | TNFAIP3 |
| ALK | CD101 | DNMT1 | GNAS | KMT2B | NRAS | SF3A | TNFSF13B |
| APC | CDC27 | DNMT3A | HAVCR2 | KMT2C | NSD2 | SF3B1 | TP53 |
| ARID1A | CDKN2A | EGFR | IDH1 | KMT2D | PDGFRB | SH2B3 | TPMT |
| ARID2A | CEBPA | EP300 | IDH2 | KMT2E | PHF6 | SOCS1 | TRAF3 |
| ASXL1 | CREBBP | EPHA7 | IKZF1 | KIT | PRDM1 | STAG2 | U2AF1 |
| ATM | CRLF2 | ETNK1 | IL7R | KRAS | PTEN | STAT3 | UBA2 |
| ATRX | CSF3R | ETV6 | IL3RA | KZF1 | PTPN11 | STAT5a | WT1 |
| BCL2 | CUX1 | EZH2 | IRF4 | MECOM | PIGA | SUZ12 | XPO1 |
| BCL6 | CXCL12 | FAM46C | IRF6 | MN1 | RELN | TAL1 | ZEB2 |
| BCOR | CXCR4 | FAT1 | JAK1 | MPL | RUNX1 | TCF3 | ZRSR2 |
| BCORL1 | CYLD | FBXW7 | JAK2 | MYC | SAMHD1 | TERC |  |

Table S4. The distribution of gene mutations in different groups of IPSS-R.

| **Gene** | **Very low**  **(n=7)** | **Low**  **(n=38)** | **Intermediate**  **(n=66)** | **High**  **(n=106)** | **Very high**  **(n=87)** |
| --- | --- | --- | --- | --- | --- |
| TET2(n=83) | 2(2.41%) | 14(16.87%) | 15(18.07%) | 23(27.71%) | 29(34.94%) |
| TP53(n=53) | 0(0.00%) | 7(13.21%) | 6(11.32%) | 9(16.98%) | 31(58.49%) |
| TET1(n=51) | 0(0.00%) | 3(5.88%) | 7(13.73%) | 22(43.14%) | 19(37.25%) |
| ASXL1(n=51) | 0(0.00%) | 11(21.57%) | 5(9.80%) | 23(45.10%) | 12(23.53%) |
| EZH2(n=48) | 2(4.17%) | 1(2.08%) | 14(29.17%) | 20(41.67%) | 11(22.92%) |
| FAT1(n=44) | 2(4.55%) | 8(18.18%) | 10(22.73%) | 9(20.45%) | 15(34.09%) |
| EP300(n=40) | 0(0.00%) | 5(12.50%) | 14(35.00%) | 12(30.00%) | 9(22.50%) |
| DDX18(n=35) | 2(5.71%) | 3(8.57%) | 9(25.71%) | 8(22.86%) | 13(37.14%) |
| CD101(n=32) | 0(0.00%) | 5(15.63%) | 8(25.00%) | 10(31.25%) | 9(28.13%) |
| SF3B1(n=31) | 2(6.45%) | 5(16.13%) | 9(29.03%) | 9(29.03%) | 6(19.35%) |
| RUNX1(n=30) | 0(0.00%) | 2(6.67%) | 2(6.67%) | 12(40.00%) | 14(46.67%) |
| U2AF1(n=29) | 2(6.90%) | 4(13.79%) | 9(31.03%) | 11(37.93%) | 3(10.34%) |
| DNMT1(n=27) | 0(0.00%) | 3(11.11%) | 9(33.33%) | 7(25.93%) | 8(29.63%) |
| DNMT3A(n=23) | 1(4.35%) | 0(0.00%) | 7(30.43%) | 12(52.17%) | 3(13.04%) |
| KDM6A(n=20) | 0(0.00%) | 0(0.00%) | 6(30.00%) | 12(60.00%) | 2(10.00%) |
| IL7R(n=19) | 2(10.53%) | 5(26.32%) | 3(15.79%) | 3(15.79%) | 6(31.58%) |
| BCOR(n=19) | 0(0.00%) | 4(21.05%) | 6(31.58%) | 9(47.37%) | 0(0.00%) |
| SH2B3(n=17) | 0(0.00%) | 5(29.41%) | 0(0.00%) | 8(47.06%) | 4(23.53%) |
| IL3RA(n=17) | 0(0.00%) | 0(0.00%) | 6(35.29%) | 6(35.29%) | 5(29.41%) |
| CEBPA(n=16) | 0(0.00%) | 2(12.50%) | 3(18.75%) | 9(56.25%) | 2(12.50%) |
| BCL6(n=14) | 0(0.00%) | 2(14.29%) | 5(35.71%) | 5(35.71%) | 2(14.29%) |

Table S5. The evaluation of the response to cytoruduction in MDS patients without allo-HSCT. (a) in the normal karyotypes and aberrant karyotypes, respectively; (b) in different gene mutation status (mutation frequency ≥5%).

**a**

| **Response** | **Normal Karyotypes** | **Aberrant Karyotypes** | **P** |
| --- | --- | --- | --- |
|  | **(N=51) (no, %)** | **(N=44) (no, %)** |  |
| CR | 20(39.2%) | 11(25%) | 0.595 |
| PR | 8(15.7%) | 6(13.6%) |  |
| HI | 4(7.8%) | 4(9.1%) |  |
| SD | 5(9.8%) | 6(13.6%) |  |
| PD | 14(27.5%) | 17(38.6%) |  |

*CR*, complete response (including marrow CR); *PR*, partial response; *HI*, hematological improvement; *SD*, stable disease; *PD*, progressive disease.

**b**

| **Gene** | **Mutation status** | **Overall response** | | **P** |
| --- | --- | --- | --- | --- |
|  |  | **Yes** | **No** |  |
| TET2 | Mutated(n=17) | 6(35.3%) | 11(64.7%) | 0.060 |
|  | Wild-type(n=78) | 47(60.3%) | 31(39.7%) |  |
| TP53 | Mutated(n=28) | 9(32.1%) | 19(67.9%) | 0.003 |
|  | Wild-type(n=67) | 44(65.7%) | 23(34.3%) |  |
| TET1 | Mutated(n=6) | 4(66.7%) | 2(33.3%) | 0.691 |
|  | Wild-type(n=89) | 49(55.1%) | 40(44.9%) |  |
| ASXL1 | Mutated(n=9) | 4(44.4%) | 5(55.6%) | 0.502 |
|  | Wild-type(n=86) | 49(57%) | 37(43%) |  |
| EZH2 | Mutated(n=13) | 10(76.9%) | 3(23.1%) | 0.099 |
|  | Wild-type(n=82) | 43(52.4%) | 39(47.6%) |  |
| FAT1 | Mutated(n=17) | 10(58.8%) | 7(41.2%) | 0.781 |
|  | Wild-type(n=78) | 43(55.1%) | 35(44.9%) |  |
| EP300 | Mutated(n=13) | 12(92.3%) | 1(7.7%) | 0.004 |
|  | Wild-type(n=82) | 41(50.0%) | 41(50.0%) |  |
| DDX18 | Mutated(n=4) | 1(25%) | 3(75%) | 0.318 |
|  | Wild-type(n=91) | 52(57.1%) | 39(42.9%) |  |
| CD101 | Mutated(n=11) | 7(63.6%) | 4(36.4%) | 0.750 |
|  | Wild-type(n=84) | 46(54.8%) | 38(45.2%) |  |
| SF3B1 | Mutated(n=14) | 6(42.9%) | 8(57.1%) | 0.291 |
|  | Wild-type(n=81) | 47(58%) | 34(42%) |  |
| RUNX1 | Mutated(n=7) | 3(42.9%) | 4(57.1%) | 0.696 |
|  | Wild-type(n=88) | 50(56.8%) | 38(43.2%) |  |
| U2AF1 | Mutated(n=16) | 10(62.5%) | 6(37.5%) | 0.553 |
|  | Wild-type(n=79) | 43(54.4%) | 36(45.6%) |  |
| DNMT1 | Mutated(n=9) | 8(88.9%) | 1(11.1%) | 0.073 |
|  | Wild-type(n=86) | 45(52.3%) | 41(47.7%) |  |
| DNMT3A | Mutated(n=10) | 6(60%) | 4(40%) | 1.000 |
|  | Wild-type(n=85) | 47(55.3%) | 38(44.7%) |  |
| KDM6A | Mutated(n=2) | 0(0%) | 2(100%) | 0.193 |
|  | Wild-type(n=93) | 53(57%) | 40(43%) |  |
| IL7R | Mutated(n=6) | 1(16.7%) | 5(83.3%) | 0.084 |
|  | Wild-type(n=89) | 52(58.4% | 37(41.6%) |  |
| BCOR | Mutated(n=6) | 3(50%) | 3(50%) | 1.000 |
|  | Wild-type(n=89) | 50(56.2%) | 39(43.8%) |  |
| SH2B3 | Mutated(n=7) | 3(42.9%) | 4(57.1%) | 0.696 |
|  | Wild-type(n=88) | 50(56.8%) | 38(43.2%) |  |
| IL3RA | Mutated(n=4) | 4(100%) | 0(0%) | 0.127 |
|  | Wild-type(n-91) | 49(53.8%) | 42(46.2%) |  |
| CEBPA | Mutated(n=1) | 1(100%) | 0(0%) | 1.000 |
|  | Wild-type(n=94) | 52(55.3%) | 42(44.7%) |  |
| BCL6 | Mutated(n=1) | 1(100%) | 0(0%) | 1.000 |
|  | Wild-type(n=94) | 52(55.3%) | 42(44.7%) |  |

*Overall reponse*: CR+PR+HI.

Table S6. Cox regression analysis for the the overall survival and time to leukemia transformation.

| **Variable** | **Overall Survival** | | | | | | **Time to leukemia transformation** | | | | | |
| --- | --- | --- | --- | --- | --- | --- | --- | --- | --- | --- | --- | --- |
|  | **Univariable** | | | **Multivariable** | | | **Univariable** | | | **Multivariable** | | |
|  | **HR** | **95%CI** | **P** | **HR** | **95%CI** | **P** | **HR** | **95%CI** | **P** | **HR** | **95%CI** | **P** |
| **The Whole Cohort** |  |  |  |  |  |  |  |  |  |  |  |  |
| Age | 1.02 | 1.01-1.04 | 0.001 | 1.02 | 1.00-1.03 | 0.027 | 0.99 | 0.97-0.01 | 0.299 | - | - | - |
| IPSS-R | 1.67 | 1.37-2.02 | <0.0001 | 1.80 | 1.48-2.18 | <0.0001 | 1.65 | 1.14-2.39 | 0.008 | 1.53 | 1.07-2.19 | 0.021 |
| Allo-HSCT | 0.56 | 0.38-0.81 | 0.002 | 0.50 | 0.32-0.76 | 0.001 | - | - | - | - | - | - |
| TP53 | 3.21 | 2.20-4.67 | <0.0001 | 2.36 | 1.58-3.54 | <0.0001 | 0.72 | 0.35-1.49 | 0.377 | - | - | - |
| TET2 | 1.40 | 0.96-2.05 | 0.084 | 1.78 | 0.87-3.64 | 0.113 | 2.01 | 1.07-3.78 | 0.031 | 1.78 | 0.87-3.64 | 0.113 |
| DNMT3A | 2.03 | 1.18-3.48 | 0.010 | 1.83 | 1.02-3.29 | 0.044 | 1.27 | 0.50-3,24 | 0.615 | - | - | - |
| U2AF1 | 0.47 | 0.22-1.01 | 0.053 | 0.68 | 0.31-1.49 | 0.336 | 0.74 | 0.26-2.07 | 0.561 | - | - | - |
| DDX18 | 0.89 | 0.49-1.62 | 0.705 | - | - | - | 2.63 | 1.08-6.40 | 0.033 | 2.67 | 1.03-6.93 | 0.043 |
| TET1 | 1.30 | 0.84-2.01 | 0.238 | - | - | - | 2.09 | 1.04-4.19 | 0.039 | 1.38 | 0.62-3.07 | 0.430 |
| IL-7R | 1.35 | 0.71-2.58 | 0.361 | - | - | - | 3.53 | 0.82-15.28 | 0.092 | 3.01 | 0.57-15.86 | 0.193 |
| **Normal Karyotype Group** |  |  |  |  |  |  |  |  |  |  |  |  |
| Age | 2.38 | 1.36-4.17 | 0.002 | 1.04 | 1.01-1.07 | 0.012 | 0.98 | 0.95-1.01 | 0.219 | - | - | - |
| IPSS-R | 1.24 | 1.03-1.49 | 0.023 | 1.54 | 1.14-2.08 | 0.005 | 1.68 | 0.95-3.00 | 0.077 | 1.29 | 0.55-3.01 | 0.558 |
| Allo-HSCT | 0.49 | 0.26-0.91 | 0.023 | 0.52 | 0.28-0.97 | 0.040 | - | - | - | - | - | - |
| FAT1 | 1.97 | 1.06-3.63 | 0.031 | 2.32 | 1.15-4.69 | 0.019 | 1.21 | 0.53-2.74 | 0.649 | - | - | - |
| DNMT3A | 3.79 | 1.94-7.41 | <0.0001 | 3.32 | 1.41-7.82 | 0.006 | 1.50 | 0.35-6.49 | 0.585 | - | - | - |
| IL-7R | 3.46 | 1.54-7.78 | 0.003 | 4.35 | 1.73-10.92 | 0.002 | 3.70 | 0.80-17.05 | 0.093 | 2.89 | 0.39-21.34 | 0.298 |
| EZH2 | 1.97 | 1.09-3.55 | 0.024 | 1.41 | 0.72-2.77 | 0.317 | 2.39 | 1.00-5.72 | 0.051 | 1.57 | 0.50-4.91 | 0.438 |
| TET2 | 1.70 | 0.92-3.16 | 0.092 | 2.03 | 0.99-4.16 | 0.530 | 4.00 | 1.06-15.09 | 0.040 | 5.55 | 1.13-27.18 | 0.034 |
| U2AF1 | 0.28 | 0.07-1.17 | 0.080 | 0.53 | 0.12-2.28 | 0.395 | 0.49 | 0.11-2.12 | 0.341 | - | - | - |
| TP53 | 2.34 | 1.10-4.97 | 0.028 | 2.49 | 1.09-5.70 | 0.030 | 0.27 | 0.06-1.19 | 0.083 | 0.39 | 0.07-2.30 | 0.299 |
| TET1 | 1.16 | 0.58-2.31 | 0.680 | - | - | - | 2.39 | 1.00--5.72 | 0.051 | 1.88 | 0.56-6.30 | 0.304 |
| **Aberrant Karyotype Group** |  |  |  |  |  |  |  |  |  |  |  |  |
| Age | 1.04 | 1.01-1.07 | 0.011 | 1.02 | 1.00-1.03 | 0.036 | 0.99 | 0.96-1.02 | 0.446 | - | - | - |
| IPSS-R | 1.89 | 1.40-2.57 | <0.0001 | 1.80 | 1.31-2.49 | <0.0001 | 2.40 | 1.24-4.63 | 0.009 | 1.94 | 0.87-4.34 | 0.106 |
| Allo-HSCT | 0.49 | 0.31-0.79 | 0.003 | 0.37 | 0.21-0.63 | <0.0001 | - | - | - | - | - | - |
| TP53 | 3.30 | 2.08-5.23 | <0.0001 | 2.13 | 1.26-3.59 | 0.005 | 1.54 | 0.61-3.88 | 0.365 | - | - | - |
| SF3B1 | 0.37 | 0.15-0.92 | 0.032 | 0.69 | 0.27-1.76 | 0.443 | 2.06 | 0.58-7.29 | 0.264 | - | - | - |
| RUNX1 | 0.30 | 0.07-1.24 | 0.097 | 0.37 | 0.09-1.52 | 0.166 | 3.30 | 1.02-10.71 | 0.047 | 5.43 | 1.46-20.18 | 0.012 |
| U2AF1 | 0.680 | 0.28-1.70 | 0.412 | - | - | - | 4.18 | 0.84-20.67 | 0.080 | 17.53 | 2.26-135.83 | 0.006 |
| DDX18 | 0.980 | 0.45-2.14 | 0.957 | - | - | - | 4.80 | 1.15-20.10 | 0.032 | 9.03 | 1.52-53.67 | 0.016 |
| EP300 | 0.670 | 0.32-1.39 | 0.283 | - | - | - | 0.18 | 0.06-0.58 | 0.064 | 0.40 | 0.09-1.76 | 0.226 |
| DNMT1 | 1.020 | 0.47-2.23 | 0.953 | - | - | - | 0.16 | 0.02-1.23 | 0.078 | 0.38 | 0.04-3.49 | 0.390 |

Table S7. The comparison of leukemic transformation. (a) in different groups of IPSS-R; (b) in any two groups of IPSS-R.

**a**

| **IPSS-R** | **Leukemia transformation** | | **P** |
| --- | --- | --- | --- |
|  | **YES** | **NO** |  |
| Very low (n=7) | 0(0.0%) | 7(100%) | 0.003 |
| Low (n=38) | 2(5.3%) | 36(94.7%) |  |
| Intermediate (n=66) | 8(12.1%) | 58(87.9%) |  |
| High (n=106) | 26(24.5%) | 80(75.5%) |  |
| Very high (n=87) | 26(29.9%) | 61(70.1%) |  |

**b**

| **IPSS-R** | **p** |
| --- | --- |
| **Very low(n=7)** |  |
| Low(n=38) | 1.000 |
| Intermediate(n=66) | 1.000 |
| High(n=106) | 0.349 |
| Very high(n=87) | 0.184 |
| **Low(n=38)** |  |
| Intermediate(n=66) | 0.319 |
| High(n=106) | 0.009 |
| Very high(n=87) | 0.002 |
| **Intermediate(n=66)** |  |
| High(n=106) | 0.047 |
| Very high(n=87) | 0.011 |
| **High(n=106)** |  |
| Very high(n=87) | 0.404 |

Table S8. The causes of death.

| **Causes** | **Normal Karyotypes**  **(N=52) (no, %)** | **Aberrant Karyotypes**  **(N=77) (no, %)** | **P** |
| --- | --- | --- | --- |
| Disease progression | 18 (34.6%) | 29 (37.7%) | 0.735 |
| Infections | 10 (19.2%) | 13 (16.9%) |  |
| Bleeding | 6 (11.5%) | 12 (15.6%) |  |
| Multiple organ failure | 4 (7.7%) | 9 (11.7%) |  |
| Transplantation-related complications | 4 (7.7%) | 6 (7.8%) |  |
| Others | 10(19.2%) | 8(10.4%) |  |

Table S9.  Details of genetic mutations that included in the multivariable COX analysis for the the OS and time to leukemia transformation.

| **Gene** | **Type of mutations** | **Exon** | **Base and aminoacid changes** | **VAF** |
| --- | --- | --- | --- | --- |
| TP53 | missense | 3 | c.91G>A(p.V31I) | 48.97 |
| TP53 | stop gain | 8 | c.916C>T(p.R306X) | 77.02 |
| TP53 | missense | 8 | c.857A>G(p.E286G) | 79.77 |
| TP53 | missense | 8 | c.853G>A(p.E285K) | 18.92 |
| TP53 | missense | 8 | c.853G>A(p.E285K) | 20.76 |
| TP53 | missense | 8 | c.844C>T(p.R282W) | 53.08 |
| TP53 | missense | 8 | c.844C>T(p.R282W) | 51.18 |
| TP53 | missense | 8 | c.833C>G (p.P278R) | 50.12 |
| TP53 | missense | 8 | c.833C>G (p.P278R) | 45.76 |
| TP53 | missense | 8 | c.833C>G (p.P278R) | 39.22 |
| TP53 | missense | 8 | c.818G>T(p.R273L) | 7.50 |
| TP53 | missense | 8 | c.818G>T(p.R273L) | 10.20 |
| TP53 | missense | 8 | c.818G>A(p.R273H) | 54.20 |
| TP53 | missense | 8 | c.818G>A(p.R273H) | 56.10 |
| TP53 | missense | 8 | c.818G>A(p.R273H) | 54.32 |
| TP53 | missense | 7 | c.742C>T(p.R248W) | 61.54 |
| TP53 | missense | 7 | c.733G>T(p.G245C) | 55.37 |
| TP53 | missense | 7 | c.733G>A(p.G245S) | 65.32 |
| TP53 | missense | 7 | c.733G>A(p.G245S) | 70.84 |
| TP53 | missense | 7 | c.733G>A(p.G245S) | 62.43 |
| TP53 | nonframeshift | 7 | c.717_719del(p.239_240del) | 2.83 |
| TP53 | missense | 7 | c.713G>A(p.C238Y) | 74.47 |
| TP53 | missense | 7 | c.713G>A(p.C238Y) | 71.42 |
| TP53 | missense | 7 | c.712T>A(p.C238S) | 93.03 |
| TP53 | missense | 7 | c.706T>C(p.Y236H) | 46.34 |
| TP53 | missense | 7 | c.706T>C(p.Y236H) | 53.25 |
| TP53 | missense | 6 | c.659A>G(p.Y220C) | 5.53 |
| TP53 | missense | 6 | c.659A>G(p.Y220C) | 14.87 |
| TP53 | missense | 6 | c.659A>G(p.Y220C) | 53.60 |
| TP53 | missense | 6 | c.659A>G(p.Y220C) | 63.15 |
| TP53 | missense | 6 | c.659A>G(p.Y220C) | 60.08 |
| TP53 | frameshift | 6 | c.636delT(p.F212fs) | 27.94 |
| TP53 | frameshift | 6 | c.636delT(p.F212fs) | 30.21 |
| TP53 | stop gain | 6 | c.602T>A(p.L201X) | 60.07 |
| TP53 | missense | 5 | c.527G>A(p.C176Y) | 75.90 |
| TP53 | missense | 5 | c.499C>T(p.Q167Ter) | 58.91 |
| TP53 | missense | 5 | c.490A>G(p.K164E) | 4.51 |
| TP53 | missense | 5 | c.490A>G(p.K164E) | 8.60 |
| TP53 | missense | 5 | c.455C>G(p.P152R） | 31.23 |
| TP53 | missense | 5 | c.455C>G(p.P152R） | 20.67 |
| TP53 | missense | 5 | c.455C>G(p.P152R） | 43.21 |
| TP53 | missense | 5 | c.412G>C(p.A138P) | 20.76 |
| TP53 | missense | 5 | c.412G>C(p.A138P) | 18.48 |
| TP53 | missense | 5 | c.395A>T(p.K132M) | 65.22 |
| TP53 | missense | 5 | c.395A>T(p.K132M) | 68.87 |
| TP53 | missense | 4 | c.338T>( p.F113S) | 83.61 |
| TP53 | missense | 4 | c.217G>C(p.V73L) | 11.03 |
| TP53 | missense | 4 | c.217G>C(p.V73L) | 15.30 |
| TP53 | missense | 4 | c.215C>G(p.P72R) | 52.07 |
| TP53 | missense | 4 | c.215C>G(p.P72R) | 52.09 |
| TP53 | missense | 4 | c.215C>G(p.P72R) | 52.55 |
| TP53 | missense | 4 | c.215C>G(p.P72R) | 50.99 |
| TP53 | missense | 4 | c.215C>G(p.P72R) | 87.30 |
| TET2 | missense | 3 | c.652G>A(p.V218M) | 50.32 |
| TET2 | missense | 3 | c.652G>A(p.V218M) | 49.67 |
| TET2 | missense | 3 | c.652G>A(p.V218M) | 54.91 |
| TET2 | missense | 3 | c.652G>A(p.V218M) | 53.56 |
| TET2 | missense | 3 | c.652G>A(p.V218M) | 53.46 |
| TET2 | missense | 3 | c.652G>A(p.V218M) | 48.28 |
| TET2 | missense | 3 | c.652G>A(p.V218M) | 37.20 |
| TET2 | missense | 3 | c.652G>A(p.V218M) | 26.27 |
| TET2 | missense | 3 | c.652G>A(p.V218M) | 23.28 |
| TET2 | missense | 3 | c.652G>A(p.V218M) | 49.39 |
| TET2 | missense | 3 | c.652G>A(p.V218M) | 52.03 |
| TET2 | missense | 3 | c.652G>A(p.V218M) | 50.09 |
| TET2 | missense | 3 | c.652G>A(p.V218M) | 48.29 |
| TET2 | missense | 3 | c.652G>A(p.V218M) | 49.28 |
| TET2 | missense | 3 | c.652G>A(p.V218M) | 47.29 |
| TET2 | missense | 3 | c.598C>T(p.L200F) | 19.73 |
| TET2 | missense | 11 | c.5611A>G(p.l1871V) | 41.20 |
| TET2 | missense | 11 | c.5284A>G(p.I1762V) | 49.07 |
| TET2 | missense | 11 | c.5284A>G(p.I1762V) | 51.43 |
| TET2 | missense | 11 | c.5284A>G(p.I1762V) | 45.42 |
| TET2 | missense | 11 | c.5284A>G(p.I1762V) | 81.89 |
| TET2 | missense | 11 | c.5284A>G(p.I1762V) | 49.64 |
| TET2 | missense | 11 | c.5284A>G(p.I1762V) | 51.31 |
| TET2 | missense | 11 | c.5284A>G(p.I1762V) | 49.73 |
| TET2 | missense | 11 | c.5284A>G(p.I1762V) | 72.89 |
| TET2 | missense | 11 | c.5284A>G(p.I1762V) | 51.25 |
| TET2 | missense | 11 | c.5284A>G(p.I1762V) | 50.72 |
| TET2 | missense | 11 | c.5284A>G(p.I1762V) | 50.71 |
| TET2 | missense | 11 | c.5284A>G(p.I1762V) | 49.92 |
| TET2 | missense | 11 | c.5284A>G(p.I1762V) | 49.91 |
| TET2 | missense | 11 | c.5284A>G(p.I1762V) | 49.59 |
| TET2 | missense | 11 | c.5284A>G(p.I1762V) | 49.13 |
| TET2 | missense | 11 | c.5284A>G(p.I1762V) | 48.33 |
| TET2 | missense | 11 | c.5284A>G(p.I1762V) | 48.16 |
| TET2 | missense | 11 | c.5284A>G(p.I1762V) | 48.02 |
| TET2 | missense | 11 | c.5284A>G(p.I1762V) | 43.57 |
| TET2 | missense | 11 | c.5284A>G(p.I1762V) | 50.40 |
| TET2 | missense | 11 | c.5162T>G(p.L1721W) | 51.09 |
| TET2 | missense | 11 | c.5162T>G(p.L1721W) | 66.88 |
| TET2 | missense | 11 | c.5162T>G(p.L1721W) | 49.87 |
| TET2 | missense | 11 | c.5162T>G(p.L1721W) | 52.72 |
| TET2 | missense | 11 | c.5162T>G(p.L1721W) | 52.96 |
| TET2 | missense | 11 | c.5162T>G(p.L1721W) | 50.89 |
| TET2 | missense | 11 | c.5162T>G(p.L1721W) | 49.04 |
| TET2 | missense | 11 | c.5162T>G(p.L1721W) | 48.20 |
| TET2 | missense | 11 | c.5162T>G(p.L1721W) | 45.46 |
| TET2 | missense | 11 | c.5162T>G(p.L1721W) | 50.69 |
| TET2 | missense | 11 | c.4931C>T(p.P1644L) | 48.05 |
| TET2 | missense | 11 | c.4667C>T(p.S1556F) | 12.93 |
| TET2 | missense | 11 | c.4667C>T(p.S1556F) | 15.05 |
| TET2 | missense | 11 | c.4667C>T(p.S1556F) | 13.33 |
| TET2 | missense | 11 | c.4667C>T(p.S1556F) | 12.31 |
| TET2 | missense | 11 | c.4667C>T(p.S1556F) | 12.29 |
| TET2 | missense | 11 | c.4667C>T(p.S1556F) | 11.84 |
| TET2 | missense | 9 | c.4160A>G(p.N1387S) | 3.50 |
| TET2 | missense | 9 | c.4160A>G(p.N1387S) | 1.70 |
| TET2 | missense | 6 | c.3782G>A(p.R1261H) | 2.22 |
| TET2 | missense | 6 | c.3755T>A(p.L1252Q) | 3.60 |
| TET2 | missense | 6 | c.3755T>A(p.L1252Q) | 5.20 |
| TET2 | stop gain | 6 | c.3646C>T(p.R1216X) | 43.52 |
| TET2 | missense | 5 | c.3572A>G(p.Q1191R) | 20.00 |
| TET2 | stop gain | 4 | c.3430G>T(p.E1144X) | 30.52 |
| TET2 | stop gain | 4 | c.3430G>T(p.E1144X) | 35.12 |
| TET2 | frameshift | 3 | c.3138_3139insTp.Thr1047fs） | 51.84 |
| TET2 | missense | 3 | c.3116C>T(p.S1039L) | 53.15 |
| TET2 | missense | 3 | c.3116C>T(p.S1039L) | 50.86 |
| TET2 | missense | 3 | c.3116C>T(p.S1039L) | 53.96 |
| TET2 | missense | 3 | c.3116C>T(p.S1039L) | 52.01 |
| TET2 | missense | 3 | c.3116C>T(p.S1039L) | 49.48 |
| TET2 | missense | 3 | c.3116C>T(p.S1039L) | 51.23 |
| TET2 | stop gain | 3 | c.2746C>T(p.Q916X) | 1.76 |
| TET2 | stop gain | 3 | c.2665A>T(p.K889X) | 1.70 |
| TET2 | stop gain | 3 | c.2665A>T(p.K889X) | 2.30 |
| TET2 | missense | 3 | c.2662C>T(p.Q888Ter) | 40.37 |
| TET2 | missense | 3 | c.2604T>G(p.F868L) | 46.92 |
| TET2 | missense | 3 | c.2440C>T(p.R814C) | 50.42 |
| TET2 | frameshift | 3 | c.2264dupA(p.E755fs) | 47.09 |
| TET2 | frameshift | 3 | c.2069delA(p.Q690fs) | 47.56 |
| TET2 | frameshift | 3 | c.1961delA(p.Q654fs) | 1.30 |
| TET2 | stop gain | 3 | c.1259C>G(p.S420X) | 18.67 |
| TET2 | stop gain | 3 | c.1259C>G(p.S420X) | 20.31 |
| TET2 | frameshift | 3 | c.1237delC(p.P413fs) | 47.81 |
| TET2 | frameshift | 3 | c.1237delC(p.P413fs) | 43.28 |
| DNMT3A | missense | 7 | c.802G>A(p.D268N) | 34.78 |
| DNMT3A | missense | 7 | c.802G>A(p.D268N) | 42.39 |
| DNMT3A | missense | 7 | c.802G>A(p.D268N) | 46.57 |
| DNMT3A | missense | 7 | c.802G>A(p.D268N) | 35.93 |
| DNMT3A | missense | 7 | c.802G>A(p.D268N) | 48.23 |
| DNMT3A | missense | 7 | c.802G>A(p.D268N) | 50.03 |
| DNMT3A | missense | 23 | c.2645G>A(p.R882H) | 45.24 |
| DNMT3A | missense | 23 | c.2645G>A(p.R882H) | 43.09 |
| DNMT3A | missense | 23 | c.2645G>A(p.R882H) | 39.87 |
| DNMT3A | missense | 23 | c.2645G>A(p.R882H) | 45.66 |
| DNMT3A | missense | 23 | c.2645G>A(p.R882H) | 42.23 |
| DNMT3A | missense | 23 | c.2645G>A(p.R882H) | 37.59 |
| DNMT3A | missense | 23 | c.2645G>A(p.R882H) | 40.89 |
| DNMT3A | missense | 23 | c.2645G>A(p.R882H) | 29.27 |
| DNMT3A | missense | 19 | c.2264T>C(p.F755S) | 5.35 |
| DNMT3A | missense | 19 | c.2264T>C(p.F755S) | 2.23 |
| DNMT3A | missense | 19 | c.2264T>C(p.F755S) | 15.93 |
| DNMT3A | missense | 19 | c.2264T>C(p.F755S) | 3.53 |
| DNMT3A | missense | 19 | c.2264T>C(p.F755S) | 21.15 |
| DNMT3A | frameshift | 15 | c.1711delG(p.A571fs) | 1.15 |
| DNMT3A | frameshift | 14 | c.1645dupT(p.C549fs) | 34.5 |
| DNMT3A | stop gain | 14 | c.1560C>A(p.C520X) | 2.45 |
| DNMT3A | stop gain | 14 | c.1560C>A(p.C520X) | 1.77 |
| U2AF1 | missense | 6 | c.470A>G(p.Q157R) | 29.20 |
| U2AF1 | missense | 6 | c.470A>G(p.Q157R) | 25.33 |
| U2AF1 | missense | 6 | c.470A>C(p.Q157P) | 42.78 |
| U2AF1 | missense | 6 | c.470A>C(p.Q157P) | 26.06 |
| U2AF1 | missense | 6 | c.470A>C(p.Q157P) | 25.54 |
| U2AF1 | missense | 6 | c.470A>C(p.Q157P) | 19.77 |
| U2AF1 | missense | 6 | c.470A>C(p.Q157P) | 21.13 |
| U2AF1 | missense | 6 | c.248G>A, c.467G>A(p.R83H, p.R156H) | 18.11 |
| U2AF1 | missense | 2 | c.101C>T(p.S34F) | 17.90 |
| U2AF1 | missense | 2 | c.101C>T(p.S34F) | 23.23 |
| U2AF1 | missense | 2 | c.101C>T(p.S34F) | 20.68 |
| U2AF1 | missense | 2 | c.101C>T(p.S34F) | 44.70 |
| U2AF1 | missense | 2 | c.101C>T(p.S34F) | 36.64 |
| U2AF1 | missense | 2 | c.101C>T(p.S34F) | 30.09 |
| U2AF1 | missense | 2 | c.101C>T(p.S34F) | 44.10 |
| U2AF1 | missense | 2 | c.101C>T(p.S34F) | 27.15 |
| U2AF1 | missense | 2 | c.101C>T(p.S34F) | 24.20 |
| U2AF1 | missense | 2 | c.101C>T(p.S34F) | 25.60 |
| U2AF1 | missense | 2 | c.101C>T(p.S34F) | 32.50 |
| U2AF1 | missense | 2 | c.101C>T(p.S34F) | 20.43 |
| U2AF1 | missense | 2 | c.101C>T(p.S34F) | 41.13 |
| U2AF1 | missense | 2 | c.101C>T(p.S34F) | 29.07 |
| U2AF1 | missense | 2 | c.101C>A(p.S34Y) | 38.99 |
| U2AF1 | missense | 2 | c.101C>A(p.S34Y) | 37.87 |
| U2AF1 | missense | 2 | c.101C>A(p.S34Y) | 35.59 |
| U2AF1 | missense | 2 | c.101C>A(p.S34Y） | 35.10 |
| U2AF1 | missense | 2 | c.101C>A(p.S34Y） | 36.54 |
| U2AF1 | missense | 2 | c.101C>A(p.S34Y） | 32.24 |
| U2AF1 | missense | 2 | c.101C>A(p.S34Y） | 54.22 |
| DDX18 | missense | 6 | c.793A>G(p.M265V) | 45.93 |
| DDX18 | missense | 6 | c.793A>G(p.M265V) | 53.87 |
| DDX18 | missense | 4 | c.548G>A(p.C183Y) | 46.04 |
| DDX18 | missense | 4 | c.548G>A(p.C183Y) | 41.35 |
| DDX18 | missense | 4 | c.548G>A(p.C183Y) | 46.19 |
| DDX18 | missense | 2 | c.281C>G(p.T94S) | 54.85 |
| DDX18 | missense | 2 | c.281C>G(p.T94S) | 49.41 |
| DDX18 | missense | 2 | c.281C>G(p.T94S) | 47.21 |
| DDX18 | missense | 2 | c.281C>G(p.T94S) | 54.76 |
| DDX18 | missense | 2 | c.281C>G(p.T94S) | 46.35 |
| DDX18 | missense | 2 | c.281C>G(p.T94S) | 55.24 |
| DDX18 | missense | 2 | c.281C>G(p.T94S) | 53.25 |
| DDX18 | missense | 2 | c.281C>G(p.T94S) | 50.77 |
| DDX18 | missense | 2 | c.281C>G(p.T94S) | 50.74 |
| DDX18 | missense | 2 | c.281C>G(p.T94S) | 50.54 |
| DDX18 | missense | 2 | c.281C>G(p.T94S) | 50.4 |
| DDX18 | missense | 2 | c.281C>G(p.T94S) | 49.45 |
| DDX18 | missense | 2 | c.281C>G(p.T94S) | 47.68 |
| DDX18 | missense | 2 | c.281C>G(p.T94S) | 48.34 |
| DDX18 | missense | 2 | c.281C>G(p.T94S) | 45.14 |
| DDX18 | missense | 2 | c.281C>G(p.T94S) | 48.21 |
| DDX18 | missense | 2 | c.281C>G(p.T94S) | 46.36 |
| DDX18 | missense | 2 | c.281C>G(p.T94S) | 45.40 |
| DDX18 | missense | 2 | c.281C>G(p.T94S) | 40.60 |
| DDX18 | missense | 2 | c.281C>G(p.T94S) | 45.07 |
| DDX18 | missense | 2 | c.281C>G(p.T94S) | 43.88 |
| DDX18 | missense | 2 | c.281C>G(p.T94S) | 42.32 |
| DDX18 | missense | 2 | c.281C>G(p.T94S) | 49.27 |
| DDX18 | missense | 2 | c.281C>G(p.T94S) | 50.76 |
| DDX18 | missense | 2 | c.281C>G(p.T94S) | 53.22 |
| DDX18 | nonframeshift | 2 | c.227_229delAAG p.Glu76del | 48.19 |
| DDX18 | nonframeshift | 2 | c.227_229delAAG p.Glu76del | 52.09 |
| DDX18 | nonframeshift | 2 | c.227_229delAAG p.Glu76del | 48.3 |
| DDX18 | nonframeshift | 2 | c.227_229delAAG p.Glu76del | 45.76 |
| DDX18 | missense | 10 | c.1386T>G(p.N462K) | 48.12 |
| TET1 | missense | 2 | c.767C>T(p.A256V) | 53.58 |
| TET1 | missense | 2 | c.767C>T(p.A256V) | 50.44 |
| TET1 | missense | 2 | c.767C>T(p.A256V) | 43.47 |
| TET1 | missense | 2 | c.767C>T(p.A256V) | 46.65 |
| TET1 | missense | 2 | c.767C>T(p.A256V) | 52.29 |
| TET1 | missense | 2 | c.767C>T(p.A256V) | 52.04 |
| TET1 | missense | 2 | c.767C>T(p.A256V) | 50.58 |
| TET1 | missense | 2 | c.767C>T(p.A256V) | 45.79 |
| TET1 | missense | 2 | c.767C>T(p.A256V) | 36.29 |
| TET1 | missense | 2 | c.767C>T(p.A256V) | 44.57 |
| TET1 | missense | 2 | c.767C>T(p.A256V) | 46.21 |
| TET1 | missense | 2 | c.767C>T(p.A256V) | 47.29 |
| TET1 | missense | 2 | c.577T>A(p.S193T) | 44.32 |
| TET1 | missense | 2 | c.577T>A(p.S193T) | 40.98 |
| TET1 | missense | 2 | c.577T>A(p.S193T) | 48.68 |
| TET1 | missense | 2 | c.577T>A(p.S193T) | 53.08 |
| TET1 | missense | 2 | c.577T>A(p.S193T) | 48.83 |
| TET1 | missense | 2 | c.577T>A(p.S193T) | 52.84 |
| TET1 | missense | 2 | c.577T>A(p.S193T) | 52.54 |
| TET1 | missense | 2 | c.577T>A(p.S193T) | 51.03 |
| TET1 | missense | 2 | c.577T>A(p.S193T) | 49.92 |
| TET1 | missense | 2 | c.577T>A(p.S193T) | 49.07 |
| TET1 | missense | 2 | c.577T>A(p.S193T) | 48.58 |
| TET1 | missense | 2 | c.577T>A(p.S193T) | 46.70 |
| TET1 | missense | 2 | c.577T>A(p.S193T) | 46.19 |
| TET1 | missense | 2 | c.577T>A(p.S193T) | 22.10 |
| TET1 | missense | 2 | c.577T>A(p.S193T) | 59.11 |
| TET1 | missense | 2 | c.577T>A(p.S193T) | 47.55 |
| TET1 | missense | 2 | c.577T>A(p.S193T) | 56.26 |
| TET1 | missense | 2 | c.577T>A(p.S193T) | 50.12 |
| TET1 | missense | 2 | c.577T>A(p.S193T) | 38.05 |
| TET1 | missense | 2 | c.518T>C(p.I173T) | 46.97 |
| TET1 | missense | 2 | c.518T>C(p.I173T) | 40.88 |
| TET1 | missense | 2 | c.485A>G(p.D162G) | 51.54 |
| TET1 | missense | 2 | c.485A>G(p.D162G) | 49.74 |
| TET1 | missense | 2 | c.485A>G(p.D162G) | 52.02 |
| TET1 | missense | 2 | c.485A>G(p.D162G) | 50.00 |
| TET1 | missense | 2 | c.1460C>T(p.S487L) | 47.78 |
| TET1 | missense | 2 | c.1460C>T(p.S487L) | 49.05 |
| TET1 | missense | 2 | c.1460C>T(p.S487L) | 50.54 |
| TET1 | missense | 2 | c.1460C>T(p.S487L) | 45.3 |
| TET1 | missense | 2 | c.1460C>T(p.S487L) | 48.1 |
| TET1 | missense | 2 | c.1460C>T(p.S487L) | 47.32 |
| TET1 | missense | 2 | c.1460C>T(p.S487L) | 47.56 |
| TET1 | missense | 2 | c.1460C>T(p.S487L) | 47.3 |
| TET1 | missense | 2 | c.1460C>T(p.S487L) | 44.35 |
| TET1 | missense | 2 | c.1460C>T(p.S487L) | 45.83 |
| TET1 | missense | 2 | c.1460C>T(p.S487L) | 39.08 |
| TET1 | missense | 2 | c.1460C>T(p.S487L) | 45.32 |
| TET1 | missense | 2 | c.1460C>T(p.S487L) | 46.43 |
| TET1 | missense | 2 | c.1460C>T(p.S487L) | 49.32 |
| IL-7R | missense | 6 | c.731C>T(p.T244I) | 37.53 |
| IL-7R | missense | 6 | c.731C>T(p.T244I) | 47.98 |
| IL-7R | missense | 6 | c.731C>T(p.T244I) | 57.77 |
| IL-7R | missense | 6 | c.731C>T(p.T244I) | 52.95 |
| IL-7R | missense | 6 | c.731C>T(p.T244I) | 46.13 |
| IL-7R | missense | 6 | c.731C>T(p.T244I) | 51.13 |
| IL-7R | missense | 6 | c.731C>T(p.T244I) | 51.02 |
| IL-7R | missense | 6 | c.731C>T(p.T244I) | 50.32 |
| IL-7R | missense | 6 | c.731C>T(p.T244I) | 39.98 |
| IL-7R | missense | 6 | c.731C>T(p.T244I) | 49.65 |
| IL-7R | missense | 6 | c.731C>T(p.T244I) | 48.73 |
| IL-7R | missense | 6 | c.731C>T(p.T244I) | 49.51 |
| IL-7R | missense | 6 | c.731C>T(p.T244I) | 47.32 |
| IL-7R | missense | 6 | c.731C>T(p.T244I) | 48.64 |
| IL-7R | missense | 6 | c.731C>T(p.T244I) | 46.53 |
| IL-7R | missense | 6 | c.731C>T(p.T244I) | 45.97 |
| IL-7R | missense | 6 | c.731C>T(p.T244I) | 48.13 |
| IL-7R | missense | 6 | c.731C>T(p.T244I) | 47.63 |
| IL-7R | missense | 6 | c.731C>T(p.T244I) | 51.94 |
| FAT1 | missense | 10 | c.5770G>A(p.G1924R) | 51.23 |
| FAT1 | missense | 3 | c.3337G>A(p.D1113N) | 48.93 |
| FAT1 | missense | 25 | c.12695A>G(p.Y4232C) | 50.14 |
| FAT1 | missense | 10 | c.6822C>G(p.D2274E) | 52.05 |
| FAT1 | missense | 10 | c.6822C>G(p.D2274E) | 49.94 |
| FAT1 | missense | 10 | c.6822C>G(p.D2274E) | 44.27 |
| FAT1 | missense | 10 | c.6822C>G(p.D2274E) | 50.98 |
| FAT1 | missense | 10 | c.6822C>G(p.D2274E) | 45.21 |
| FAT1 | missense | 10 | c.6822C>G(p.D2274E) | 55.13 |
| FAT1 | missense | 10 | c.6822C>G(p.D2274E) | 53.42 |
| FAT1 | missense | 10 | c.6822C>G(p.D2274E) | 53.16 |
| FAT1 | missense | 10 | c.6822C>G(p.D2274E) | 54.55 |
| FAT1 | missense | 10 | c.6822C>G(p.D2274E) | 52.18 |
| FAT1 | missense | 10 | c.6822C>G(p.D2274E) | 49.87 |
| FAT1 | missense | 10 | c.6822C>G(p.D2274E) | 51.03 |
| FAT1 | missense | 10 | c.6822C>G(p.D2274E) | 47.63 |
| FAT1 | missense | 10 | c.6822C>G(p.D2274E) | 50.15 |
| FAT1 | missense | 10 | c.6822C>G(p.D2274E) | 48.42 |
| FAT1 | missense | 10 | c.6822C>G(p.D2274E) | 43.12 |
| FAT1 | missense | 10 | c.6822C>G(p.D2274E) | 47.73 |
| FAT1 | missense | 10 | c.6822C>G(p.D2274E) | 77.89 |
| FAT1 | missense | 10 | c.6822C>G(p.D2274E) | 67.98 |
| FAT1 | missense | 10 | c.6822C>G(p.D2274E) | 51.19 |
| FAT1 | missense | 10 | c.6822C>G(p.D2274E) | 49.10 |
| FAT1 | missense | 10 | c.6822C>G(p.D2274E) | 54.86 |
| FAT1 | missense | 10 | c.6822C>G(p.D2274E) | 49.65 |
| FAT1 | missense | 2 | c.1842C>G(p.F614L) | 47.65 |
| FAT1 | missense | 2 | c.1842C>G(p.F614L) | 46.23 |
| FAT1 | missense | 2 | c.1444G>A(p.V482I) | 49.27 |
| FAT1 | missense | 2 | c.1444G>A(p.V482I) | 49.63 |
| FAT1 | missense | 2 | c.1444G>A(p.V482I) | 48.83 |
| FAT1 | missense | 2 | c.1444G>A(p.V482I) | 47.38 |
| FAT1 | missense | 2 | c.1444G>A(p.V482I) | 43.12 |
| FAT1 | missense | 2 | c.1444G>A(p.V482I) | 50.96 |
| FAT1 | missense | 2 | c.1444G>A(p.V482I) | 46.44 |
| FAT1 | missense | 2 | c.1444G>A(p.V482I) | 51.22 |
| FAT1 | missense | 2 | c.1444G>A(p.V482I) | 50.70 |
| FAT1 | missense | 2 | c.1444G>A(p.V482I) | 43.87 |
| FAT1 | missense | 22 | c.12008T>C(p.V4003A) | 14.21 |
| FAT1 | missense | 22 | c.12008T>C(p.V4003A) | 12.36 |
| FAT1 | missense | 22 | c.11818A>G(p.T3940A) | 48.36 |
| FAT1 | missense | 22 | c.11818A>G(p.T3940A) | 52.30 |
| FAT1 | missense | 22 | c.11818A>G(p.T3940A) | 48.74 |
| FAT1 | missense | 22 | c.11818A>G(p.T3940A) | 44.15 |
| EZH2 | stop gain | 8 | c.895A>T(p.K299X) | 2.83 |
| EZH2 | stop gain | 8 | c.895A>T(p.K299X) | 5.43 |
| EZH2 | stop gain | 8 | c.895A>T(p.K299X) | 23.13 |
| EZH2 | stop gain | 8 | c.895A>T(p.K299X) | 18.76 |
| EZH2 | frameshift | 7 | c.728delA(p.K243fs) | 43.72 |
| EZH2 | missense | 6 | c.553G>C(p.D185H) | 46.43 |
| EZH2 | missense | 6 | c.553G>C(p.D185H) | 45.53 |
| EZH2 | missense | 6 | c.553G>C(p.D185H) | 49.08 |
| EZH2 | missense | 6 | c.553G>C(p.D185H) | 47.67 |
| EZH2 | missense | 6 | c.553G>C(p.D185H) | 34.11 |
| EZH2 | missense | 6 | c.553G>C(p.D185H) | 50.81 |
| EZH2 | missense | 6 | c.553G>C(p.D185H) | 50.81 |
| EZH2 | missense | 6 | c.553G>C(p.D185H) | 50.62 |
| EZH2 | missense | 6 | c.553G>C(p.D185H) | 52.61 |
| EZH2 | missense | 6 | c.553G>C(p.D185H) | 46.77 |
| EZH2 | missense | 6 | c.553G>C(p.D185H) | 53.75 |
| EZH2 | missense | 6 | c.553G>C(p.D185H) | 49.98 |
| EZH2 | missense | 6 | c.553G>C(p.D185H) | 53.31 |
| EZH2 | missense | 6 | c.553G>C(p.D185H) | 51.62 |
| EZH2 | missense | 6 | c.553G>C(p.D185H) | 46.92 |
| EZH2 | missense | 6 | c.553G>C(p.D185H) | 51.30 |
| EZH2 | missense | 6 | c.553G>C(p.D185H) | 49.25 |
| EZH2 | missense | 6 | c.553G>C(p.D185H) | 50.83 |
| EZH2 | missense | 6 | c.553G>C(p.D185H) | 5106 |
| EZH2 | missense | 6 | c.553G>C(p.D185H) | 49.39 |
| EZH2 | missense | 6 | c.553G>C(p.D185H) | 46.98 |
| EZH2 | missense | 6 | c.553G>C(p.D185H) | 49.23 |
| EZH2 | missense | 6 | c.553G>C(p.D185H) | 50.10 |
| EZH2 | missense | 6 | c.553G>C(p.D185H) | 49.08 |
| EZH2 | missense | 6 | c.553G>C(p.D185H) | 43.21 |
| EZH2 | missense | 6 | c.553G>C(p.D185H) | 48.93 |
| EZH2 | missense | 6 | c.553G>C(p.D185H) | 47.77 |
| EZH2 | missense | 6 | c.553G>C(p.D185H) | 4654 |
| EZH2 | missense | 6 | c.553G>C(p.D185H) | 44.04 |
| EZH2 | missense | 6 | c.553G>C(p.D185H) | 43.71 |
| EZH2 | missense | 6 | c.553G>C(p.D185H) | 40.92 |
| EZH2 | missense | 6 | c.553G>C(p.D185H) | 30.91 |
| EZH2 | missense | 6 | c.553G>C(p.D185H) | 25.99 |
| EZH2 | missense | 6 | c.553G>C(p.D185H) | 65.43 |
| EZH2 | missense | 5 | c.395C>T(p.P1321L) | 20.21 |
| EZH2 | frameshift |  | c.2228dupG(p.G743fs) | 44.00 |
| EZH2 | missense | 19 | c.2139A>G(p.I713M) | 3.40 |
| EZH2 | missense | 19 | c.2139A>G(p.I713M) | 1.42 |
| EZH2 | missense | 18 | c.2050C>T(p.R684C) | 8.94 |
| EZH2 | missense | 18 | c.2050C>T(p.R684C) | 6.08 |
| EZH2 | stop gain | 17 | c.1957C>T(p.Q653X) | 6.78 |
| EZH2 | stop gain | 17 | c.1957C>T(p.Q653X) | 2.59 |
| EZH2 | frameshift | 3 | c.163delA p.Ile55fs | 32.27 |
| SF3B1 | missense | 19 | c.2719G>T(p.D907Y) | 6.76 |
| SF3B1 | missense | 4 | c.2602G>A(p.D868N) | 54.99 |
| SF3B1 | missense | 16 | c.2342A>G(p.D781G) | 34.88 |
| SF3B1 | missense | 16 | c.2342A>G(p.D781G) | 44.81 |
| SF3B1 | missense | 16 | c.2342A>G(p.D781G) | 37.61 |
| SF3B1 | missense | 16 | c.2225G>A(p.G742D) | 35.12 |
| SF3B1 | missense | 16 | c.2225G>A(p.G742D) | 33.96 |
| SF3B1 | missense | 15 | c.2218G>A(p.G740R) | 21.11 |
| SF3B1 | missense | 15 | c.2218G>A(p.G740R) | 19.03 |
| SF3B1 | missense | 15 | c.2098A>G(p.K700E) | 8.28 |
| SF3B1 | missense | 15 | c.2098A>G(p.K700E) | 15.17 |
| SF3B1 | missense | 15 | c.2098A>G(p.K700E) | 46.87 |
| SF3B1 | missense | 15 | c.2098A>G(p.K700E) | 30.11 |
| SF3B1 | missense | 15 | c.2098A>G(p.K700E) | 34.15 |
| SF3B1 | missense | 15 | c.2098A>G(p.K700E) | 45.00 |
| SF3B1 | missense | 15 | c.2098A>G(p.K700E) | 48.33 |
| SF3B1 | missense | 15 | c.2098A>G(p.K700E) | 46.53 |
| SF3B1 | missense | 15 | c.2098A>G(p.K700E) | 41.04 |
| SF3B1 | missense | 15 | c.2098A>G(p.K700E) | 20.65 |
| SF3B1 | missense | 15 | c.2098A>G(p.K700E) | 24.33 |
| SF3B1 | missense | 15 | c.2098A>G(p.K700E) | 19.12 |
| SF3B1 | missense | 15 | c.2098A>G(p.K700E) | 20.80 |
| SF3B1 | missense | 15 | c.2098A>G(p.K700E) | 17.99 |
| SF3B1 | missense | 15 | c.2098A>G(p.K700E) | 16.54 |
| SF3B1 | missense | 15 | c.2098A>G(p.K700E) | 43.12 |
| SF3B1 | missense | 15 | c.2098A>G(p.K700E) | 40.00 |
| SF3B1 | missense | 15 | c.2098A>G(p.K700E) | 24.65 |
| SF3B1 | missense | 14 | c.1998G>T(p.K666N) | 45.98 |
| SF3B1 | missense | 14 | c.1998G>T(p.K666N) | 46.44 |
| SF3B1 | missense | 14 | c.1998G>T(p.K666N) | 47.39 |
| SF3B1 | missense | 14 | c.1873C>T(p.R625C) | 27.72 |
| RUNX1 | missense | 6 | c.611G>A(p.R204Q) | 31.60 |
| RUNX1 | missense | 6 | c.611G>A(p.R204Q) | 5.73 |
| RUNX1 | missense | 5 | c.497G>A(p.R166Q) | 36.15 |
| RUNX1 | missense | 5 | c.497G>A(p.R166Q) | 26.75 |
| RUNX1 | missense | 5 | c.497G>A(p.R166Q) | 28.20 |
| RUNX1 | missense | 5 | c.497G>A(p.R166Q) | 29.15 |
| RUNX1 | missense | 5 | c.497G>A(p.R166Q) | 32.97 |
| RUNX1 | missense | 5 | c.497G>A(p.R166Q) | 28.12 |
| RUNX1 | missense | 5 | c.497G>A(p.R166Q) | 32.54 |
| RUNX1 | nonframeshift | 5 | c.354_355insTGTCCTTTGACTGGTGTTTAGGTGp.Val119Cys | 21.07 |
| RUNX1 | missense | 4 | c.320G>A(p.R107H) | 27.16 |
| RUNX1 | missense | 4 | c.319C>T(p.R107C) | 10.9 |
| RUNX1 | missense | 4 | c.319C>T(p.R107C) | 8.99 |
| RUNX1 | frameshift | 4 | c.296_297insTG p.Ser100AlafsTer23 | 46.15 |
| RUNX1 | missense | 15 | c.2098A>G(p.K700E) | 8.28 |
| RUNX1 | missense | 4 | c.136G>A p.Ala46Thr | 57.50 |
| RUNX1 | missense | 4 | c.136G>A p.Ala46Thr | 36.74 |
| RUNX1 | frameshift |  | c.1277_1278insCCCCCCCCCC(p. P426fs) | 6.30 |
| RUNX1 | frameshift | 9 | c.1210dupC(p.H404fs) | 31.16 |
| RUNX1 | missense | 9 | c.1190A>G(p.Q397R) | 45.23 |
| RUNX1 | missense | 9 | c.1190A>G(p.Q397R) | 47.65 |
| RUNX1 | missense | 9 | c.1190A>G(p.Q397R) | 49.88 |
| RUNX1 | missense | 9 | c.1190A>G(p.Q397R) | 49.31 |
| RUNX1 | missense | 9 | c.1190A>G(p.Q397R) | 51.59 |
| RUNX1 | missense | 9 | c.1190A>G(p.Q397R) | 51.40 |
| RUNX1 | missense | 9 | c.1190A>G(p.Q397R) | 49.18 |
| RUNX1 | frameshift | 9 | c.1103_1104insTp.Met368fs | 52.49 |
| RUNX1 | missense | 9 | c.1030G>A(p.D344N) | 52.75 |
| RUNX1 | frameshift | 9 | c.1023dupC(p.I342fs) | 41.94 |
| RUNX1 | frameshift | 9 | c. 1062_1063insCCCGCA TGCACTATCCAGGC GCCTTCACC(p. Y355fs) | 1.01 |
| EP300 | missense | 4 | c.923C>T(p.P308L) | 87.8 |
| EP300 | missense | 31 | c.6400A>G(p.N2134D) | 23.13 |
| EP300 | missense | 31 | c.6400A>G(p.N2134D) | 25.33 |
| EP300 | missense | 31 | c.6400A>G(p.N2134D) | 27.21 |
| EP300 | missense | 31 | c.6400A>G(p.N2134D) | 16.03 |
| EP300 | missense | 31 | c.6400A>G(p.N2134D) | 19.87 |
| EP300 | missense | 31 | c.6400A>G(p.N2134D) | 15.18 |
| EP300 | missense | 31 | c.6400A>G(p.N2134D) | 17.43 |
| EP300 | missense | 31 | c.6400A>G(p.N2134D) | 27.80 |
| EP300 | missense | 31 | c.6400A>G(p.N2134D) | 16.87 |
| EP300 | missense | 31 | c.6400A>G(p.N2134D) | 14.89 |
| EP300 | missense | 31 | c.6400A>G(p.N2134D) | 28.06 |
| EP300 | missense | 31 | c.6400A>G(p.N2134D) | 29.44 |
| EP300 | missense | 31 | c.6400A>G(p.N2134D) | 26.75 |
| EP300 | missense | 31 | c.6400A>G(p.N2134D) | 24.31 |
| EP300 | missense | 31 | c.6400A>G(p.N2134D) | 20.09 |
| EP300 | missense | 31 | c.6400A>G(p.N2134D) | 25.33 |
| EP300 | missense | 31 | c.6400A>G(p.N2134D) | 24.50 |
| EP300 | missense | 31 | c.6400A>G(p.N2134D) | 16.78 |
| EP300 | missense | 31 | c.6400A>G(p.N2134D) | 21.97 |
| EP300 | missense | 31 | c.6400A>G(p.N2134D) | 15.52 |
| EP300 | missense | 31 | c.6400A>G(p.N2134D) | 18.91 |
| EP300 | missense | 31 | c.6400A>G(p.N2134D) | 13.80 |
| EP300 | missense | 31 | c.6400A>G(p.N2134D) | 13.04 |
| EP300 | missense | 31 | c.6400A>G(p.N2134D) | 14.50 |
| EP300 | missense | 31 | c.6400A>G(p.N2134D) | 18.38 |
| EP300 | missense | 31 | c.6400A>G(p.N2134D) | 25.41 |
| EP300 | missense | 31 | c.6400A>G(p.N2134D) | 19.57 |
| EP300 | missense | 31 | c.6400A>G(p.N2134D) | 23.98 |
| EP300 | missense | 31 | c.6400A>G(p.N2134D) | 30.01 |
| EP300 | missense | 15 | c.2989A>G(p.I997V) | 45.49 |
| EP300 | missense | 15 | c.2989A>G(p.I997V) | 51.78 |
| EP300 | missense | 15 | c.2989A>G(p.I997V) | 50.00 |
| EP300 | missense | 15 | c.2989A>G(p.I997V) | 47.98 |
| EP300 | missense | 15 | c.2989A>G(p.I997V) | 49.71 |
| EP300 | missense | 15 | c.2989A>G(p.I997V) | 49.68 |
| EP300 | missense | 15 | c.2989A>G(p.I997V) | 48.31 |
| EP300 | missense | 15 | c.2989A>G(p.I997V) | 47.99 |
| EP300 | missense | 15 | c.2989A>G(p.I997V) | 45.52 |
| EP300 | missense | 15 | c.2989A>G(p.I997V) | 47.81 |
| DNMT1 | missense | 4 | c.358G>C(p.V120L) | 49.98 |
| DNMT1 | missense | 4 | c.358G>C(p.V120L) | 47.66 |
| DNMT1 | missense | 4 | c.358G>C(p.V120L) | 48.74 |
| DNMT1 | missense | 4 | c.358G>C(p.V120L) | 47.99 |
| DNMT1 | missense | 4 | c.290A>G(p.H97R) | 53.45 |
| DNMT1 | missense | 4 | c.290A>G(p.H97R) | 51.86 |
| DNMT1 | missense | 4 | c.290A>G(p.H97R) | 49.08 |
| DNMT1 | missense | 4 | c.290A>G(p.H97R) | 47.53 |
| DNMT1 | missense | 4 | c.290A>G(p.H97R) | 52.69 |
| DNMT1 | missense | 4 | c.290A>G(p.H97R) | 55.43 |
| DNMT1 | missense | 4 | c.290A>G(p.H97R) | 52.61 |
| DNMT1 | missense | 4 | c.290A>G(p.H97R) | 50.22 |
| DNMT1 | missense | 4 | c.290A>G(p.H97R) | 51.74 |
| DNMT1 | missense | 4 | c.290A>G(p.H97R) | 47.83 |
| DNMT1 | missense | 4 | c.290A>G(p.H97R) | 51.5 |
| DNMT1 | missense | 4 | c.290A>G(p.H97R) | 50.53 |
| DNMT1 | missense | 4 | c.290A>G(p.H97R) | 49.09 |
| DNMT1 | missense | 4 | c.290A>G(p.H97R) | 47.87 |
| DNMT1 | missense | 4 | c.290A>G(p.H97R) | 46.52 |
| DNMT1 | missense | 4 | c.290A>G(p.H97R) | 46.64 |
| DNMT1 | missense | 4 | c.290A>G(p.H97R) | 43.98 |
| DNMT1 | missense | 4 | c.290A>G(p.H97R) | 46.00 |
| DNMT1 | missense | 4 | c.290A>G(p.H97R) | 42.40 |
| DNMT1 | missense | 4 | c.290A>G(p.H97R) | 40.96 |
| DNMT1 | missense | 4 | c.290A>G(p.H97R) | 41,00 |
| DNMT1 | missense | 4 | c.290A>G(p.H97R) | 43.21 |
| DNMT1 | missense | 4 | c.290A>G(p.H97R) | 41.34 |
